# Delineating Substrate Diversity of Disparate Short-Chain Dehydrogenase Reductase from *Debaryomyces hansenii*

**DOI:** 10.1371/journal.pone.0170202

**Published:** 2017-01-20

**Authors:** Arindam Ghatak, Nagakumar Bharatham, Anirudh P. Shanbhag, Santanu Datta, Janani Venkatraman

**Affiliations:** 1 Biomoneta Research Private Limited, Bangalore, India; 2 Centre for Cellular and Molecular Platforms (C-CAMP), National Centre for Biological Sciences, Tata Institute of Fundamental Research, Bangalore, India; 3 Bugworks Research India Private Limited, Bangalore, India; 4 Department of Biophysics, Molecular Biology and Bioinformatics, University of Calcutta, Kolkata, India; Universidad de Santiago de Compostela, SPAIN

## Abstract

Short-chain dehydrogenase reductases (SDRs) have been utilized for catalyzing the reduction of many aromatic/aliphatic prochiral ketones to their respective alcohols. However, there is a paucity of data that elucidates their innate biological role and diverse substrate space. In this study, we executed an in-depth biochemical characterization and substrate space mapping (with 278 prochiral ketones) of an unannotated SDR (DHK) from *Debaryomyces hansenii* and compared it with structurally and functionally characterized SDR *Synechococcus elongatus*. PCC 7942 FabG to delineate its industrial significance. It was observed that DHK was significantly more efficient than FabG, reducing a diverse set of ketones albeit at higher conversion rates. Comparison of the FabG structure with a homology model of DHK and a docking of substrate to both structures revealed the presence of additional flexible loops near the substrate binding site of DHK. The comparative elasticity of the cofactor and substrate binding site of FabG and DHK was experimentally substantiated using differential scanning fluorimetry. It is postulated that the loop flexibility may account for the superior catalytic efficiency of DHK although the positioning of the catalytic triad is conserved.

## Introduction

Short-chain dehydrogenase reductases (SDRs) were first characterized in *Drosophila melanogaster* and were found to have similar catalytic properties like alcohol dehydrogenases (Medium Chain Dehydrogenase Reductase) albeit having lesser length. They comprise a large superfamily of lyases, isomerases, epimerases, dehydrogenases, etc. showing similar architecture viz. Rossmann folds and a conserved catalytic triad. SDRs are divided into seven major classes namely classical, extended, intermediate, complex, atypical, divergent and unknown. Although most of the dehydrogenases and reductases catalyze the same type of reaction, they are grouped in either classical or extended families and are ubiquitous in plants, fungi, and bacteria. Classical and extended SDRs include almost 30,000 members across 150 subfamilies [1,2]. With such a large sample size, it is arduous to select a suitable SDR for catalyzing a substrate of interest with an industrial endgame [3].

The exigency for SDRs with high substrate diversity has gained standing due to the demand for production of essential chemicals that are structurally dissimilar to the natural substrates of these enzymes [4]. Many enzymes show activity at room temperature and show stereo-selectivity, thus making an enzymatic conversion economical and energy efficient [5]. In 2005, the Swiss Industrial Biocatalysis Consortium (SIBC) analyzed the bio-catalytic needs of seven companies (Ciba, Givaudan, Hoff-LaRoche, SAFC, Novartis, Lonza, Syngenta), and indicated that there is a dire need for SDRs which can convert multiple prochiral ketones to their respective chiral alcohol with high efficiency (BioWorld Europe, 2005) [6,7,8].

Literature is replete with random high throughput screening of SDRs (Table 1) for identifying the ideal enzyme that can catalyze a particular substrate. In contrast, we have focused on mapping the catalytic efficiency of a diverse range of substrates to find an enzyme catering to the industrial need of manufacturing choice chiral synthons. In recent years, many new SDRs from yeasts and bacteria have been isolated and characterized. The reasoning behind these studies was based on the organism’s capability to catalyze a specific substrate of value *in vivo*. However, fungi, especially yeasts, are often present in a complex environment of cellulose, fats, as well as complex carbohydrates and are capable of metabolizing toxic compounds secreted by competing organisms. Using this dialectic, we hypothesize that a microorganism growing in a complex environment should unequivocally harbor SDRs capable of converting a diverse array of chemicals (prochiral ketones in this case). To validate this hypothesis, we have selected an unannotated SDR from *Debaryomyces hansenii*, a halotolerant food spoilage yeast which can metabolize simple carbon to complex carbohydrates [9,10].

**Table 1:** SDRs and their substrate range. Some of the reported SDRs in literature which have been tested on a limited number of substrates, thus establishing their role in the conversion of related compounds but not exploring their ability to convert a wide substrate space.

Organism	SDR	Properties	Comments
*Synechococcus elongatus*PCC 7942	FabG	Involved in reduction the of acyl group in acyl carrier protein.	Can reduce few acetophenone derivatives, β ketoesters, and aliphatic ketones.[11]
*Rhodococcus jostii* TMP1	TpdE	Involved in the catabolism of tetramethyl pyrazine.	Can reduce 2,3- and 3,4-diketones, acetoin and few of its acylated derivatives.[12]
*Candida magnoliae*	cAR	Short chain dehydrogenase participating in the reduction of xenobiotics.	Shown to catalyze common aldehydes and ketones, but is moderately specific.[13]
*Pichia stipitis*	PsCR	PsCRI and PsCRII are both carbonyl reductases which help in the metabolism of arachidonic acid (Secondary metabolite)	Few aldehyde reductions have been reported, especially acetate derivatives.[14]
*Sporobolomyces_salmonicolor*	AR II and AR III	First reported enzyme to show link between human ADR and microbial ADR (ARI) subsequently AR II and AR III were reported	Few aldehyde reductions have been reported. [15]
*Arabidopsis thaliana*	SDR1	Participates in glucose signalling and absisic acid biosynthesis	No ketoreduction reactions have been reported.[16]
*Camellia sinensis*	CsSDR	Participates in the reduction of secondary metabolites	Ketoreduction has been reported for propanoids and benzoids. Substrate space covered is limited.[17]
*Kluyveromyces lactis*	KaCR1	Carbonyl reductase which help in the metabolism of arachidonic acid (Secondary metabolite)	Shown to catalyze common aldehyde and ketones, but is highly specific and has low promiscuity.[18]
*Candida parapsilosis* CDC317	CPE	Both carbonyl reductase which help in metabolism of arachidonic acid and SDR have been reported.	Limited amount of ketoreductions have been reported.[19]
*Candida vishwanathii* MTCC 5158	Carbonyl Reductase	Carbonyl reductase which help in metabolism of arachidonic acid (Secondary metabolite)	Limited amount of ketoreductions have been reported.[20]

The current study focuses on characterizing the unannotated SDR belonging to SDR113E family from *Debaryomyces hansenii* (DHK) and mapping its ability to catalyze a various range of substrates. To further justify the industrial attribute of DHK, we chose another structurally, functionally and industrially well characterized SDR (β-keto ACP reductase or FabG) from *Synechococcus elongatus*. PCC 7942, a photoautotrophic bacteria thriving in relatively simpler fresh water environment. FabG from various species has been used previously for reducing economically viable ketones. We sought to map the diverse substrate catalytic activity of *Synechococcus elongatus* PCC7942.FabG to manifest the industrial relevance of DHK.

## Materials and Methods

### Strains and plasmid

*Escherichia coli* strain DH5α was used to prepare plasmids and BL21 (DE3) was used to over express proteins. GSure Plasmid MiniPrep kits were purchased from GCC Biotech India. DHK (Sequence ID: ref|XP_458533.2) from *Debaryomyces hansenii* and FabG (Sequence ID: ref|4DML_A) from *Synechococcus elongatus* PCC 7942 was codon optimized, synthesized and cloned into pET28a vector by GCC Biotech India. The plasmid when induced with Isopropyl-β-d-thiogalactoside (IPTG) produced DHK and FabG proteins with a hexa-histidine tag at the N-terminal.

### Chemical reagents

NADPH, NADH, Ethyl 4-chloro acetoacetate, DMSO were purchased from Sigma-Aldrich, USA, Sodium Phosphate monobasic, Sodium Phosphate dibasic, Sodium Chloride, Imidazole were purchased from Amresco, USA and was the finest grade available. All bacterial growth media and Isopropyl-β-d-thiogalactoside (IPTG) were obtained from HiMedia, India. Test molecules were procured from eMolecules Library, USA.

### Heterologous expression and gel purification of DHK and FabG

Chemically competent *E*.*coli* DH5α was transformed by pET28a-DHK and pET28a-FabG separately and selected on LB agar plate with a selection pressure of Kanamycin and used for plasmid purification. *E*.*coli* BL21 (DE3) was transformed with the purified plasmids and incubated overnight at 37°C. A single colony was picked and grown in 5ml starter culture supplemented with 50μg/ml of kanamycin. 1ml of the starter culture was used to inoculate 1000ml LB Broth supplemented with 50μg/ml of kanamycin. The bacterial growth culture was induced with 150μM of IPTG at 0.5 O.D at 600nm and incubated at 18°C post-induction overnight.

The cells were harvested by centrifugation at 4000g for 15 minutes and resuspended in lysis buffer (100mM Sodium Phosphate,100mM NaCl,10mM Imidazole, pH = 7.4). The cells were lysed on ice by sonication at 40% amplitude with ON /OFF cycle of 10 seconds for 15 rounds and the cell debris was removed by centrifugation at 22,000 g for 20 minutes. The recombinant N-terminal hexa-histidine tagged DHK was purified by Ni-NTA affinity chromatography with 4ml bed volume and eluted by using elution buffer (100mM Sodium Phosphate, 100mMNaCl, 200mM Imidazole, pH 7.4). Desalting of the protein containing buffer was done using the PD 10 column (GE health care; USA). The purity of the eluted protein fractions was determined by SDS-PAGE. In order to decipher the oligomeric state of the enzyme, it was run in a Superdex S-75 Prep column using 100mM Sodium Phosphate, 100mM NaCl pH 7.4 as a buffer.

For affinity purification of FabG, previously reported conditions were followed [11].

### Conditions of FabG activity assay

The activity of FabG was determined using 100μM Ethyl 4-chloro acetoacetate 100μM NADPH in 50mM Sodium phosphate buffer pH 8.0 as published elsewhere [11].

### Determination of optimum buffering conditions for DHK

To determine the optimum pH and the buffer concentration for maximum enzyme activity we considered three different buffers based on their buffering ability at certain pH range namely, 100mM sodium citrate (pH 4.0–6.0), 100mM sodium phosphate (pH 5.8–8.0) and 50mM Tris-HCl (pH 8.0–11.0). 1μg/ml of protein was used to catalyze 100μM of both NADPH and Ethyl 4-chloro acetoacetate.

### Optimization of pH and temperature for DHK

After determining buffering conditions, optimum pH for DHK was determined in Sodium-Phosphate (pH 5.8–8.0) and alternatively, potassium-phosphate (pH 5.8–8.0) buffers of various concentrations (20mM, 50mM, 75mM, 100mM, 120mM, 150mM, 200mM) for performing the same enzymatic reaction as mentioned previously. The optimum temperature was determined under standard reaction condition at various temperatures (20–42.5°C).

### Determination of reaction mechanism for DHK

In order to understand the reaction mechanism of DHK, the activity of the enzyme was monitored spectrophotometrically at 340nm by varying both the substrate and cofactor concentration under standard reaction conditions. The concentration of the substrate, Ethyl 4-chloro acetoacetate was varied from 100μM to 1200μM and that of the co-factor, NADPH was from 50μM to 300μM. The initial velocities of the reactions were taken into account and thereby a Lineweaver-Burk plot was made to understand whether the reaction mechanism is Ping-pong or random/compulsory ordered bi-bi reaction [21]. Differential scanning fluorimetry was done using SYPRO orange dye (Ex/Em at 300, 470/570 nm) obtained from Sigma-Aldrich. The reaction was performed in RT-PCR machine 7900HT (Applied Biosystems) using DHK (2.5μM final concentration), NAD(P)H, Ethyl 4-chloro acetoacetate and Acetophenone (50μM as final concentration). The assay was performed between 25°C to 90°C with the rate of 1°C/minute.

### Differential Scanning Fluorimetric assay for the stability of DHK and FabG

In order to check the stability of the DHK and FabG, differential scanning fluorimetric assay was done for both the proteins (2.5μM final concentration) and then 20x SYPRO orange dye was added (Ex/Em at 300, 470/570 nm) in the reaction with buffers as mentioned previously. The assay was performed between 25°C to 90°C with the ramp rate of 1°C/minute [22].

### Assay for DHK activity

The DHK activity was measured spectrophotometrically by Tecan Infinite Pro200 at 25°C by monitoring the reduction in absorbance of NADPH at 340nm (ε = 6220M^-1^cm^-1^). A standard reaction mixture consisted 100μM NADPH, 100μM Ethyl 4-chloro acetoacetate, 4% DMSO, 1% BSA and ~27nM of purified enzyme (1μgm/ml) in 100mM of Sodium-phosphate buffer (pH 7.4) in a 250μl of total reaction volume. The concentration of enzyme was determined by spectrophotometer (215/225nm method) and Bradford method [23]. To test for the enzyme’s substrate space, we screened DHK with 278 prochiral ketones from a set of 3000 in-house compound library. For every assay 100μM NADPH, 100μM of substrate, 1% BSA, 4% DMSO was used in a buffer containing 100mM sodium phosphate, 100mM NaCl at pH 7.4. In order to check for the enzyme’s ability to reduce industrially important compounds, we have used intermediate ketones of Sitagliptin, Dolastatin, Montelukast and 4-chloro benzophenone as substrates under normal enzymatic conditions.

### Determination of kinetic constants

For enzyme kinetics, thermal shift and activity assay experiments 100mM Sodium Phosphate Buffer, 100mM NaCl was used for performing initial velocity and activity studies were monitored by NADPH utilization. The kinetics of DHK (1μg/ml i.e. 25.5nM) was done by saturating the reaction mixture with one of the substrates and using various concentrations of the other substrate. 2mM Ethyl 4-chloro acetoacetate was used to determine the Km of NADPH, and 300μM NADPH was used in the reaction to determine the Km of Ethyl 4-chloro acetoacetate.

### Homology model generation for DHK

Identification of the DHK (Swiss-Prot code Q6BTD7) homolog was carried out by performing sequence database searches with protein BLAST (blastp). Best hit found in this search was used as a template for homology modeling using SWISS-MODEL web-server. The template structure selected by blastp search was specified explicitly to generate the homology models. The final 3D model was validated by PROCHECK software [24,25].

### Fingerprint based clustering and binding mode determination

Total 278 compounds were considered for MACCS [26,27] based clustering to understand ligand similarity and molecular docking study was done to understand binding orientations with catalytic site. Canvas software was used to cluster molecules based on their molecular properties which were converted to “binary fingerprints” using MACCS structural keys and bit count was calculated. Then, “hierarchical clustering” coupled with “Tanimoto similarity matrix” calculation was performed to cluster all the compounds. The ligand molecules were prepared in LigPrep module of Schrodinger suite [28] and the output was considered for multi-conformation by Macromodel [29] which uses Monte-Carlo simulation. The OPLS_2005 force field is used, and energy minimization was performed for 500 steps of the Truncated Newton Conjugate Gradient (TNCG) method. The energy window for saving structures is set to 21kJ/mol (default value). The redundancy threshold is 0.5 Å RMSD, and redundant conformers are removed. A maximum of 500 steps is allowed for the Monte Carlo sampling, and a maximum of 50 steps is allowed for the low-mode searching. A Maximum number of conformers to be generated was set to be 25. Default parameters were used for the remaining options.

Glide v 5.5 [30] molecular docking module included in Schrodinger suite was utilized to predict the binding orientations for 278 substrate molecules. In this study, we have used Glide-SP which has shown high success in earlier computational docking studies [31]. Minimization of the ligand in the field of the receptor is then carried out using the OPLS-AA force field with the default distance-dependent dielectric. The lowest energy poses are then subjected to a Monte Carlo procedure that sample nearby torsional minima and were ranked using GlideScore. Default van der Waal’s scaling was used (1.0 for the receptor and 0.8 for the ligand). Total 10000 poses (default 5000) per ligand were set for the initial phase of docking and poses per ligand per energy minimization raised to 1000 from 400. Total 10 poses per ligand were saved as output for post-docking analyses.

## Results & Discussion

### Purification of DHK and FabG

**Fig 1:**
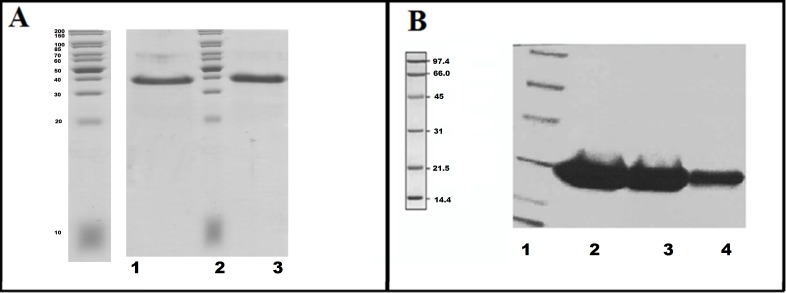
Purification of DHK and FabG. **A**. SDS-PAGE analysis of the purified DHK. Lane 1: Purified DHK through Ni-NTA, Lane 2: Puregene Broad range marker, Lane 3: Superdex S75 purified DHK. **B.** SDS-PAGE of purified FabG Lane1: Low range marker Lane 2,3,4: gel purified FabG.

The recombinant proteins with an N-terminal hexa-histidine tag were purified by standard Ni-NTA affinity chromatographic method. Samples collected at every fraction were analyzed by SDS-PAGE (4–10%). A single, thickened band corresponding to a molecular weight of ~39kDa was visualized in the final elution fraction (Fig 1A) for DHK. Simultaneous amino acid sequence analysis was carried out to calculate the molecular weight of the protein. The protein was desalted to remove the imidazole used during the purification process by a PD 10 column containing Sephadex G-25 Medium. The protein was further purified by a size exclusion chromatographic technique using HiLoad 16/600 Superdex 75 PG column (S1A Fig) and was compared with the standards of known molecular weight (Thyroglobulin, γ-globulin, Ovalbumin, Myoglobin and vitamin B12) (S1B Fig) to determine its oligomeric state. DHK, a member of the short-chain reductase family of protein, maintains a monomeric state. FabG was passed through Superdex S200 column after Ni-NTA purification and run on an SDS-PAGE (4–10%). A single, thick of ~29kDa corresponding to the purified FabG was observed (Fig 1B).

### Optimum pH and temperature

To determine the Km and Vmax of DHK, it is essential to discern the optimum temperature and pH in which enzyme was active. The activity was measured in three different buffers based on their buffering ability at certain pH range namely, 100mM sodium citrate (pH 4.0–6.0), 100mM sodium phosphate (pH 5.8–8.0) and 50mM Tris-HCl (pH 8.0–11.0) (S2A Fig) using Ethyl 4-chloro acetoacetate as the standard substrate and NADPH as the cofactor. The maximum enzymatic activity was observed at 100mM Sodium phosphate pH 7.4. A normal biased distribution in the %activity was found where the enzyme showed about ~80% of activity in buffers having a pH range of 6.8–7.6 (S2B Fig). Being an SDR in a halophyte, which subsists in coastal waters, the organism cannot survive in extreme temperatures. This was displayed when the thermal profile of the enzyme showed optimum activity at 25°C; there was a reduction in activity when it was subjected to a temperature beyond 35^0^ C (S2C Fig).

### Homology modeling of DHK

**Fig 2:**
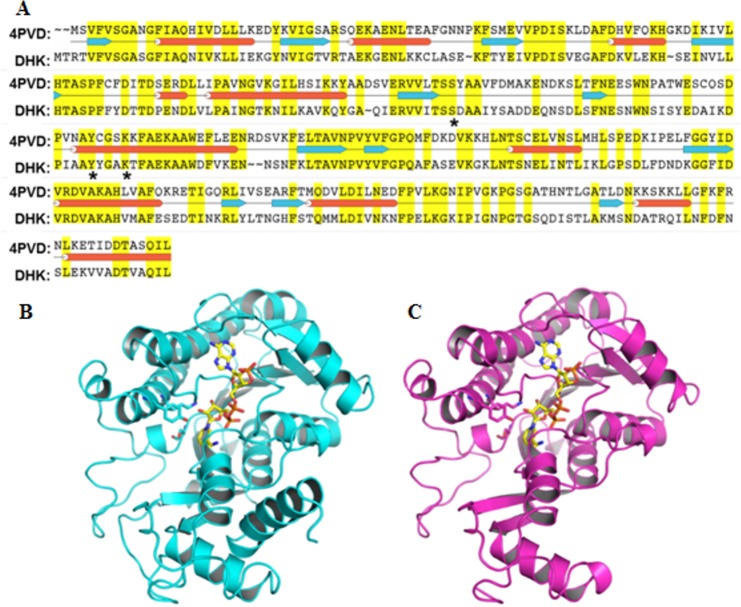
Homology model of DHK. **A.** The sequence alignment between DHK and Gre2 (4PVD) sequence which was utilized to build the homology model of DHK, **B.** Cartoon representation of template structure and **C**. DHK homology model. Co-factor NADPH represented as yellow sticks and catalytic triad highlighted with sticks in both structures.

Identification of the DHK homologues was carried out by performing sequence database searches with protein BLAST (blastp). The Protein BLAST indicated about 50% sequence identity (Fig 2A) between DHK and template protein *Saccharomyces cerevisiae* methylglyoxal/isovaleraldehyde reductase Gre2 (PDB ID: 4PVD) [32]. The homology modeling calculations were performed by SWISS-MODEL web server utilizing template structure (Fig 2B) to generate a reasonable 3D structure of DHK (Fig 2C). PROCHECK structure validation tool implemented in SWISS-MODEL web server was utilized to validate the homology modeled DHK structure. This analysis demonstrated ~89.4% of residues of the 3D structure lie in most favored regions which are comparable with template statistics i.e. ~91% residues in the most favored region. The RMSD between Cα atoms of the template and the target structures is 0.4Å. Sequence and structure comparison between template and DHK modeled structures demonstrated that the catalytic triad (serine, tyrosine, and lysine) residues which are important for ketone to alcohol reduction reaction [33] are indeed conserved in the DHK structure.

### Mechanism of enzyme action

**Fig 3:**
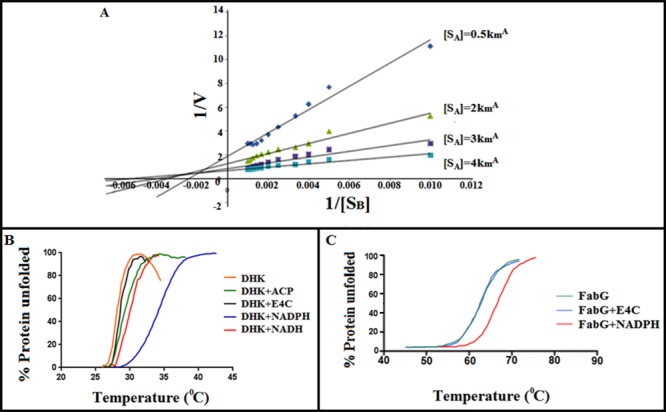
Reaction Mechanism of DHK and the effect of temperature on protein stability. **A.** The SDR from *Debaryomyces hansenii*, DHK,follows a compulsory random ordered reaction mechanism. In order to understand the reaction mechanism of DHK, the activity of the enzyme was monitored spectrophotometrically at 340nm by varying both the substrate and cofactor concentration under standard reaction conditions. The concentration of the substrate, Ethyl 4-chloro acetoacetate [S_B_] was varied from 100μM to 1200μM and that of cofactor, NADPH [S_A_] was from 50μM to 300μM. The initial velocities of the reactions were taken into account and thereby a Lineweaver-Burk graph was plotted. For a reaction mechanism to be ordered (both for compulsory as well as random) the lines in the plot was supposed to converge on the negative side. **B.** Differential scanning fluorimetry for examining the binding of cofactor and substrate with the purified enzyme. The purified enzyme has a melting temperature (Tm) of 28.04°C, when Ethyl 4-chloro acetoacetate (E4C) binds to DHK, Tm changes to 28.46°C (ΔTm = 0.42), clearly indicating that binding of E4C to the enzyme doesn’t impose stability. While when NADPH binds to DHK, Tm becomes 34.09°C (ΔTm = 6.05), giving the enzyme stability. Other cofactor, like NADH has very less affinity towards the purified protein, indicating that the enzyme prefers NADPH over NADH as a cofactor. **C.** Differential scanning fluorimetry of FabG (Tm = 58°C) with substrates NADPH (Tm = 65°C) and E4C (Tm = 59°C) (in red) and Boltzmann fit (in black).

**Fig 4:**
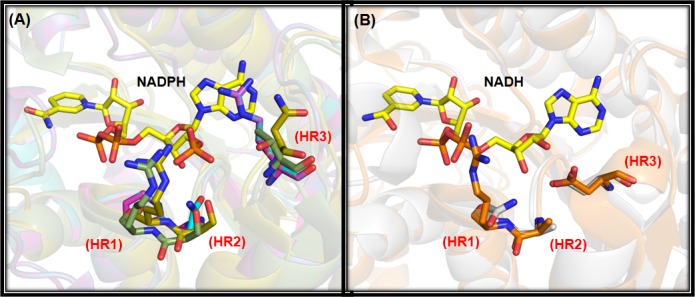
Co-factor binding pocket analyses. Comparison of NADPH/NADH binding pocket residues of (A) template structure yeast methylglyoxal/isovaleraldehyde reductase Gre2 (4PVD; Cyan colour), DHK (homology model; magenta), FabG from *Listeria monocytogenes* (4JRO; yellow), FabG from *Vibrio cholera* (4I08; green), and (B) FabG4 from *Mycobacterium tuberculosis* (3V1U; orange), FabG from *Bacillus* sp. (4NBU; gray). Three hotspot residues (HR1 to HR3) which are crucial in differentiating NADPH/NADH binding are highlighted as sticks and NADPH/NADH shown as yellow sticks in both the panels. Superimposition approach utilized to overlay the structures on template structures for comparison purpose.

We have used two different approaches to decipher the reaction mechanism of the enzyme DHK. At first, we have used a standard reaction kinetics experiment to find out whether the mechanism of reaction belongs to ping-pong or random/compulsory ordered bi-substrate reaction. Upon finding out that the reaction mechanism is compulsory/random ordered bi-bi substrate reaction (Fig 3A), we have further studied the mechanism using thermal shift assay to confer whether the mechanism belongs to compulsory or random ordered bi-substrate reaction. To portray the mechanism of DHK and its preference of co-factors (NADPH/NADH), thermal shift assay was done with the apo-enzyme and enzyme with co-factors and the substrate individually. It was observed that the apo-enzyme with NADPH has more stability than with other co-factors/substrates (NADH/ketones) which confirms the binding of NADPH (ΔTm = 6.05°C) is essential for the stability of the enzyme. NADH (ΔTm = 1.82°C) has very less affinity to the cofactor binding pocket (Fig 3B). Binding of cofactor confers stability to the enzyme, makes conformational changes in the enzyme structure and as a result the substrate gets access to the catalytic pocket. Once the ketone gets converted to the corresponding alcohol, which has a very transient binding affinity with the enzyme, it gets released from the enzyme-cofactor complex- thus making it an ordered reaction. The homology modeled DHK, template structures as well as other crystal structures (PDB ID: 4JRO and 4I08) that prefer NADPH as co-factor were compared to identify the key recognition residues at respective co-factor pockets [34, 35, 36]. All the structures were superimposed on DHK to understand such similarities. We have also considered two SDR structures (PDB ID: 3V1U and 4NBU) [37,38] which selectively accept NADH as co-factor. It was observed that three phosphate pocket hotspot residues (HR1 to HR3) are highly conserved in the template and DHK structures (Fig 4A). Comparing all the four NADPH dependent SDRs (Fig 4A) revealed that a positively charged residue is present in either HR1 or HR3 positions, whereas HR2 has a hydrophilic residue. None of these three phosphate pocket residues comprise of any negatively charged residue that are not favorable to accommodate negatively charged phosphate of NADPH. In the case of NADH dependent SDRs (Fig 4B), negatively charged residue (glutamate/aspartate) resides in HR3 whereas an alanine is present at the HR2 position. The aforementioned structural characteristics signify the sequence level co-factor selectivity of DHK towards NADPH.

### Kinetic properties of the purified enzyme

**Fig 5:**
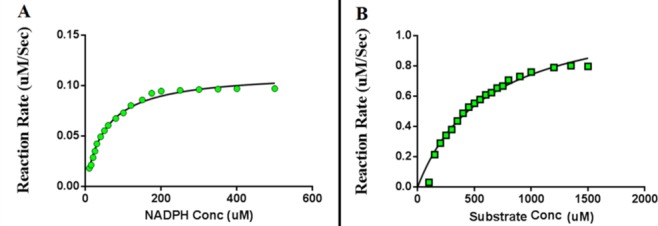
Kinectic properties of DHK. **A**. The kinetics of DHK towards NADPH with saturated amount of Ethyl 4-chloro acetoacetate. **B**. The kinetics of DHK towards Ethyl 4-chloro acetoacetate with saturated amount of NADPH.

The kinetic properties of the purified enzyme were characterized by using standard substrate (Ethyl 4-chloro acetoacetate) and co-factor (NADPH). The kinetic constants were calculated from the Michaelis-Menten’s equation using GraphPad Prism 6. The Km for NADPH was found to be 51.09μM, while that of Ethyl 4-chloro acetoacetate was 616.1 μM. The Vmax value of the purified DHK towards Ethyl 4-chloro acetoacetate was found to be 1.201μM/s/μg (Fig 5).

### Substrate diversity

**Fig 6:**
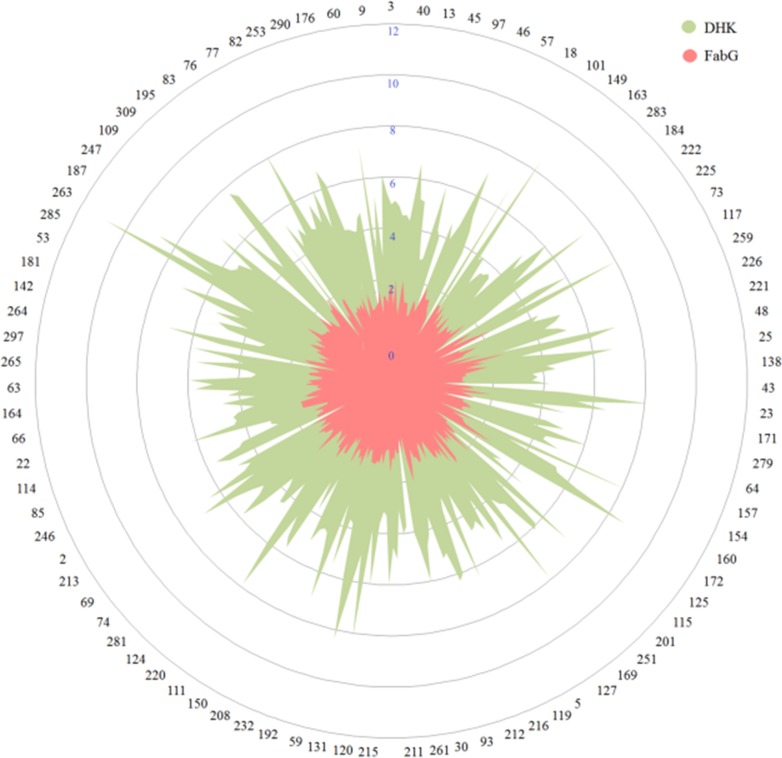
Comparison of substrate space covered by DHK and FabG. The unannotated SDR from *Debaryomyces hansenii*(DHK) can reduce prochiral ketones with higher efficiency as compared to *Synechoccus elongatus* PCC7942 ß-keto acyl carrier protein reductase (FabG), having a role in fatty acid biosynthesis. (Full list in S2 Table).

The substrate diversity of the purified enzyme was determined by using spectrophotometric, both absorbance and fluorescence assays using Ethyl 4-chloro acetoacetate as a standard substrate. A chemically diverse substrate (prochiral ketone) library consisting of 278 molecules was used to carry out the assays. These 278 compounds were selected from our internal chemical library of around 3000 molecules. Reaction rates were calculated based on the amount of cofactor (NADPH) converted per unit time, and activity table (S2 Table) was prepared. The enzyme shows the best activity with Ethyl 4-chloro acetoacetate (Fig 6). The purified enzyme didn’t show any activity in the presence of NADH. When pharmaceutical intermediate ketones were tested against DHK, it shows the ability to reduce Sitagliptin intermediate ketone and Montelukast intermediate ketone, but could not reduce Dolastatin intermediate ketone and ethyl 4-chloro benzophenone. (S1 Table)

To understand the reasons for substrate specificity and relative conversion rate differences between substrates, we have applied computational modeling methods. Initially, MACCS structural keys based clustering of the 278 substrate compounds provided a total of 83 clusters. The aim of clustering is to understand the common chemical features in substrate molecules which dictate enzyme binding and product formation. To our surprise, we observed that substrates with high relative activity and worse relative activity grouped in an identical cluster in several instances. Such exemplary substrates exhibit huge difference in conversion rates though they display minor chemical substitution differences. This observation signifies similarity based methods alone, not sufficient to predict the probable substrate molecules which can be converted by a particular enzyme. This suggests that the enzymes are sensitive to a minor modification despite having a common scaffold. To probe the differences between such exemplary compounds we have introduced molecular docking study.

**Fig 7:**
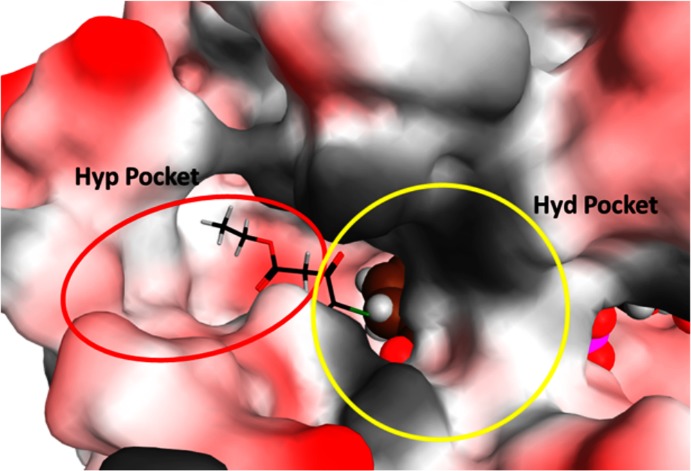
DHK substrate pocket analyses. Surface view of DHK sub-pockets (hydrophilic and hydrophobic) where substrate molecules interact with enzyme. Ethyl 4-chloro acetoacetate dock pose shown as sticks to highlight the catalytic site. NADPH molecule was highlighted with spheres.

**Fig 8:**
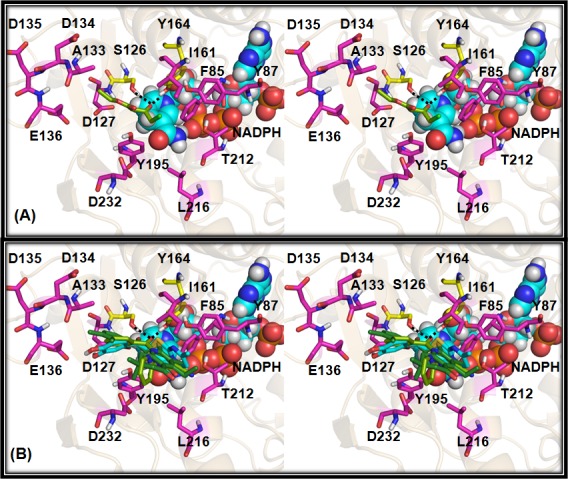
Stereo view of substrate binding modes. Molecular docking predicted binding modes of (**A**) Ethyl 4-chloro acetoacetate, (**B**) Compound 278 (dark green sticks), Compound 73 (pale green sticks) and compound 23 (cyan sticks) with DHK protein. Active site, catalytic triad residues are highlighted with magenta and yellow colour sticks respectively. Substrate molecules in (**B**) panel were superimposed. NADPH molecule shown as cyan spheres. All the residues are labelled and hydrogen bonds specified as broken lines.

The molecular docking predicted dock poses of reference and substrate molecules revealed that the key reactive carbonyl group interacts with S126 and Y164 residues of DHK. These two residues are highly conserved in all the keto-reductase enzymes. Further analyses of surrounding pocket residues revealed that the catalytic pocket could be divided into two sub-pockets. One side to the carbonyl group is made up of majorly hydrophilic residues (Hyp pocket) and other side hydrophobic (Hyd pocket) residues (Fig 7). The predicted binding mode of the reference compound, Ethyl 4-chloro acetoacetate shows that the reactive carbonyl group interacts with the catalytic residues, S126, Y164, while CH_2_Cl projecting towards Hyd pocket and other part entering into Hyp pocket (Fig 8A). Such a binding orientation carbon atom of CO group stays within 3Å distance with NADPH hydrogen atom which is crucial for reduction reaction. A 10ns duration molecular dynamics simulation of DHK:NADPH: Ethyl 4-chloro acetoacetate complex carried out to ascertain the predicted binding orientation of reference substrate molecule indeed meaningful. The backbone RMSD analyses revealed that the complex simulation was stable (S3 Fig). The substrate molecule forms 2–3 hydrogen bond interactions and stable interactions observed between Carbonyl oxygen of substrate and catalytic residues S126 and Y164 (S3B Fig). Analyses of distance between Carbonyl carbon of substrate and NADPH hydrogen which participate in reaction (S3C Fig) revealed that they are within 0.3 nm after initial equilibration period (2ns).

**Fig 9:**
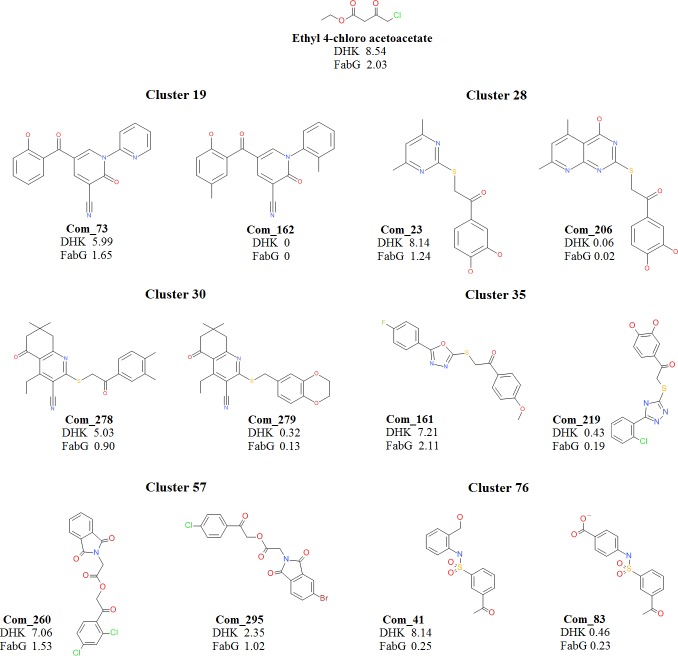
Subset of Substrate spectrum covered by DHK and FabG and their turnover by the respective enzymes. A set of 12 compounds, belonging to 6 different clusters are represented along with the standard compound (Ethyl 4-chloro acetoacetate). This is a subset of the compound library containing 278 compounds. The turnover number (Kcat) of these compounds by DHK and FabG are calculated and are represented in the figure.

Exemplary compounds represented in Fig 9 are considered as a reference set to explain the probable reasons for relative conversion rate differences based on their binding modes. Aforementioned criteria such as hydrogen bond interaction with catalytic residues (S126 and Y164) as well as within 3Å distance between carbon atom of CO group and NADPH hydrogen atom considered as prerequisite in dock pose selection. The compound 278 shows high conversion compared to closest cluster partner com279. The Com278 of cluster 30 possess two reactive carbonyl groups and the one in linker region interacts with catalytic residues (Fig 8B) while com279 lacks carbonyl moiety in the linker region. Molecular docking calculations failed to predict the probable binding orientation for Com279. The second carbonyl group present on an aliphatic ring might be responsible for the negligible conversion rate. This signifies the reactive carbonyl positioning and proper orientation is a prerequisite for optimal conversion rates. The second pair of molecules, Com73 and Com162 from cluster 19 show high and very low activity respectively. Though these two compounds have high structural similarity, replacement of ortho-pyridine ring to 2-methylphenyl may detrimental to DHK binding and product formation. We were unable to see any reasonable binding mode for Com162 which might be due to steric hindrance of a methyl group at ortho position. Based on com73 binding orientation, the ortho-pyridine ring is directed towards I161 side chain (Fig 8B) and installing methyl group at this position will clash. These compounds highlight the significance of hydrophobic residues present at the entry of the pocket.

In similar lines, Com23 from cluster 28, the hydroxyl substituted ring sits into Hyp pocket and forms hydrogen bond interactions with the D127 side chain. Second aromatic ring connected to the sulfur-containing linker is engaged with Hyd pocket (Fig 8B). Com23 had demonstrated high activity compared to reference compound while Com206 from the same cluster which is highly similar but with bicyclic ring connected to the sulfur linker, show poor activity. Molecular docking method was unable to generate any reasonable pose for this compound which could be due to steric hindrance at Hyd pocket. The experimental results confirm that Com206 is not a substrate for DHK. Other compound pairs from cluster 35 (Com161, Com219) and cluster 57 (Com260, Com295) have shown activity differences due to steric clashes with Hyd pocket residues (S4 Fig).

**Fig 10:**
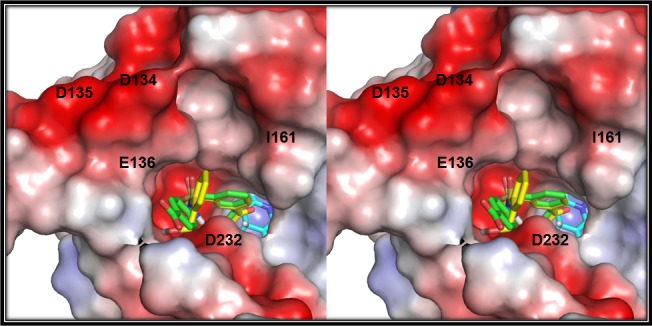
Electrostatics role in substrate binding. Stereo view of electrostatic surface of DHK withmolecular docking predicted binding modes of Compound 41 (yellow sticks) and Compound 83 (green sticks). Negatively charged residues in hydrophilic sub pockets are labelled accordingly. The NADPH molecule shown as cyan sticks.

The compounds from cluster 76 prefer to occupy Hyp pocket alone. The Com41 acetyl carbonyl interacts with catalytic residues and ortho-alcohol substituted ring interacts with the I161 side chain. The CH2OH group forms a hydrogen bond interaction D232 side chain carboxyl group. The Com41 has shown high activity compared to reference compound and Com83 which is the closest analog of an active compound has shown poor activity. This major difference in conversion rates could be attributed to charge repulsion between Hyp pocket residues and the para-carboxyl group of the compound. The Hyp pocket has around four negatively charged (D134, D135, E136, and D232) residues in the vicinity (Fig 10). Overall, these results signify steric/charge clashes with DHK pocket play a key role in substrate binding, product formation, and release.

Next we have attempted to explain probable reasons for variations in substrate conversion rates between two proteins. Structural comparison by superposition method of these two proteins revealed that most of the structured regions of proteins superimposed within 2Å backbone RMSD. Structurally conserved regions such as NADPH and catalytic triad aligned well with residual differences (S5 Fig). Major discrepancies observed at loop regions which are part of substrate binding pocket. Extended loops made up of several hydrophobic and charge residues observed in case of DHK (S5A Fig), whereas short loops present in case of FabG (S5B Fig). Such differences in substrate binding pocket might dictate substrate binding and conversion as residues on these extended loops may assist in binding and proper orientation of substrate molecules to catalytic triad. These observations were validated by performing Differential Scanning Fluorimetry (DSF) of both DHK and FabG proteins in absence and presence of co-factor (NADPH). Differential scanning fluorimetry showed that FabG is more rigid compared to DHK, wherein after binding with NADPH both enzymes display a 7°C shift which shows the protein with cofactor (holoprotein) to be more stable. However, the difference in melting temperatures between both the holoproteins is ~30°C (4B and 4C). These observations also signify DHK is more flexible compared to FabG which conforms that of FabG which is rigid and having less flexible loops [39].

## Conclusion

Our studies ascertain that the short-chain dehydrogenase reductase from *Debaryomyceshansenii*, DHK is an NADB-Rossmann oxidoreductase, and like other members of oxidoreductase family, can catalyze unfamiliar substrates. Prochiral ketone space mapping of DHK showed that it has superior catalytic efficiency compared to the industrially relevant *Synechococcus elongatus* PCC 7942 FabG.

This was further affirmed using DSF, where increased Tm indicated that FabG is considerably more rigid compared to DHK which conforms to the homology modeled DHK. We hypotheisze that this is due to presence of extra loops present near the catalytic site which enables DHK to catalyze wide variety of substrates (Loop1: VYVFGPQAFASEVKGKLNTSNELINTLIKLGPSDLFDNDKGGFID and Loop2: LTNGHFSTQMMLDIVNKNFPELKGKIPIGNPGTGSQDISTLAKMSNDATRQILNFDFNSLEKVVADTVAQILDARKRTL).

Comparison of substrate space maps of DHK with FabG showed that in ~90% of the cases, DHK showed ~5 times higher efficiency (Kcat) as compared to FabG. Also, kinetic parameters such as km for FabG against NADPH and Ethyl 4 Chloro acetoacetate is much higher than DHK (Data not shown). There have been many studies on cloning, and characterization of SDRs, from different genera, and evaluation on their ability to reduce prochiral ketones to chiral synthons. However, the selection of enzymes has been based on their role in bioconversion of secondary metabolites or participation in biochemical pathways and never guided by catalysis of diverse substrates.

Though, structural and mechanistic studies that have been carried out to delineate the dynamics of enzyme kinetics and mechanism; deciphering the substrate of an enzyme is still a challenge. Thus, it is difficult to identify an enzyme, which can reduce a novel prochiral ketone to its corresponding chiral alcohol; hence relying on substrate promiscuity of an enzyme is a safer bet. Our study has attempted to convey that SDRs, derived from organisms thriving in complex environment are more relevant for bioprospecting.

## Supporting Information

S1 Fig Oligomeric State determination of DHK. **A**. Gel purified DHK (Peak at 61.19 ml) using S75 Superdex prep grade column.**B.**Oligomeric state of DHK using S75 standards as a mode of reference for molecular weight. The protein eluted out in monomeric state.
(TIF)

S2 Fig Physico-chemical characterization of DHK. **A.** Relative activity of purified DHK in different buffer solutions (100mM sodium citrate pH 4.5–6.2, 100MM Sodium Phosphate pH 5.6–8.0 and 50mM Tris-HCl pH 7.8–11.5). The enzyme shows maximum activity at a pH of 7.4, it retains ~80% of its activity in buffer having a pH range of 6.8–8. **B.** Relative activity of DHK in 100mM sodium phosphate buffer previously shown to give optimal enzyme activity in varying pH range. **C.** The optimum temperature of the enzyme. The enzyme has highest activity at 25°C and loses most of its activity beyond 32°C.
(TIF)

S3 Fig MD simulations analyses of DHK:NADPH:Ethyl 4-chloro acetoacetate. Backbone RMSD (A), No. of hydrogen bond interactions between substrate and DHK (B) and distance between substrate Carbonyl carbon atom and NADPH hydrogen (C) shown as line graph with respective to simulation time.
(TIF)

S4 Fig Substrate binding mode prediction. Molecular docking predicted binding modes of Compound 161 (A), Compound 219 (B), Compound 260 (C) and Compound 295 (D) with DHK protein. Active site, catalytic triad residues are highlighted with magenta and yellow color sticks respectively. Substrate molecules highlighted as green sticks and NADPH shown as cyan spheres.
(TIF)

S5 Fig Comparison of DHK and FabG. Structural comparison of DHK and FabG to understand the differences in substrate binding pocket. DHK (A), FabG (B) shown as green and cyan cartoons accordingly and superposed (C). Catalytic triad shown as orange sticks.Loops highlighted as magenta and yellow color on DHK and FabG structures accordingly to highlight DHK is having relatively longer loops.
(TIF)

S1 Table Reduction of industrially important ketone by DHK. Ability of DHK to reduce industrially important ketone in-vitro. Under standard enzymatic conditions when 4 chloro benzophenone, Sitagliptin intermediate ketone, Dolastatin intermediate ketone and Montelukast intermediate ketones were used as a substrate, DHK shows the ability to reduce Sitagliptin and Montelukast intermediate ketone with a relative activity of ~70% and 90% respectively while with the other substrates it didn’t show any activity.
(XLSX)

S2 Table Comparative substrate reduction study of DHK and FabG. (XLSX)
