# Supplementary figures and images for: Delineating Substrate Diversity of Disparate Short-Chain Dehydrogenase Reductase from Debaryomyces hansenii
Source: PLoS One. 2017 Jan 20;12(1):e0170202. doi: 10.1371/journal.pone.0170202 (PMC5249140; doi:10.1371/journal.pone.0170202)

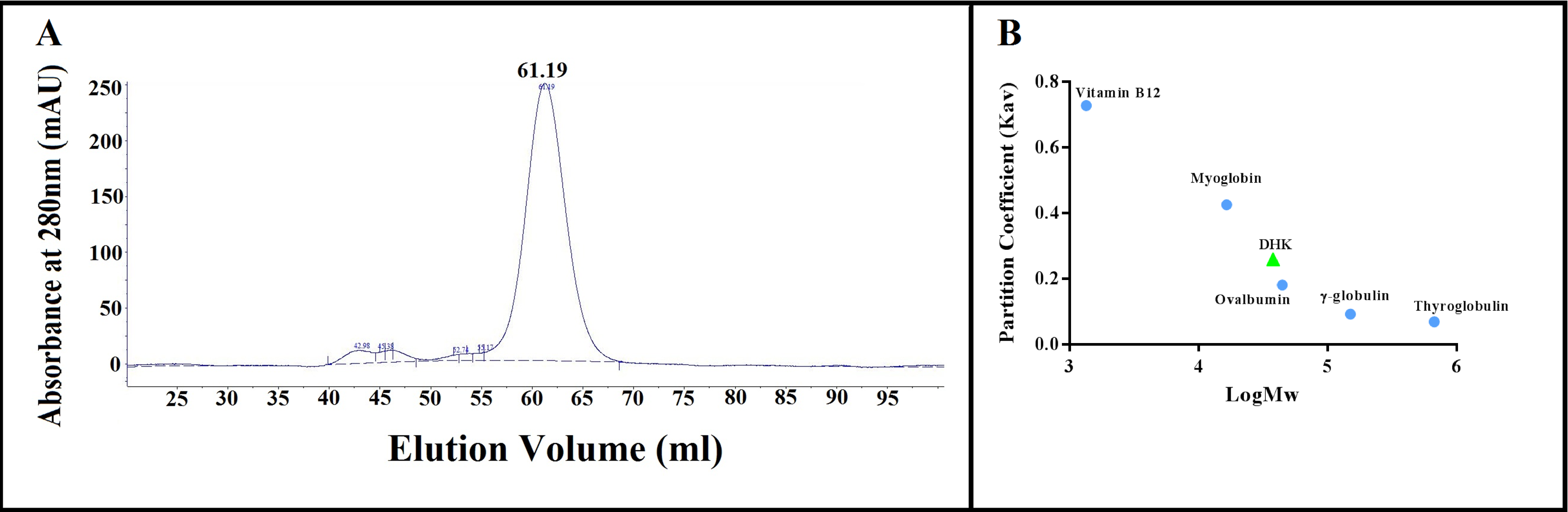

Supplement: S1 Fig — A. Gel purified DHK (Peak at 61.19 ml) using S75 Superdex prep grade column.B.Oligomeric state of DHK using S75 standards as a mode of reference for molecular weight. The protein eluted out in monomeric state. (TIF) [file pone.0170202.s001.tif]

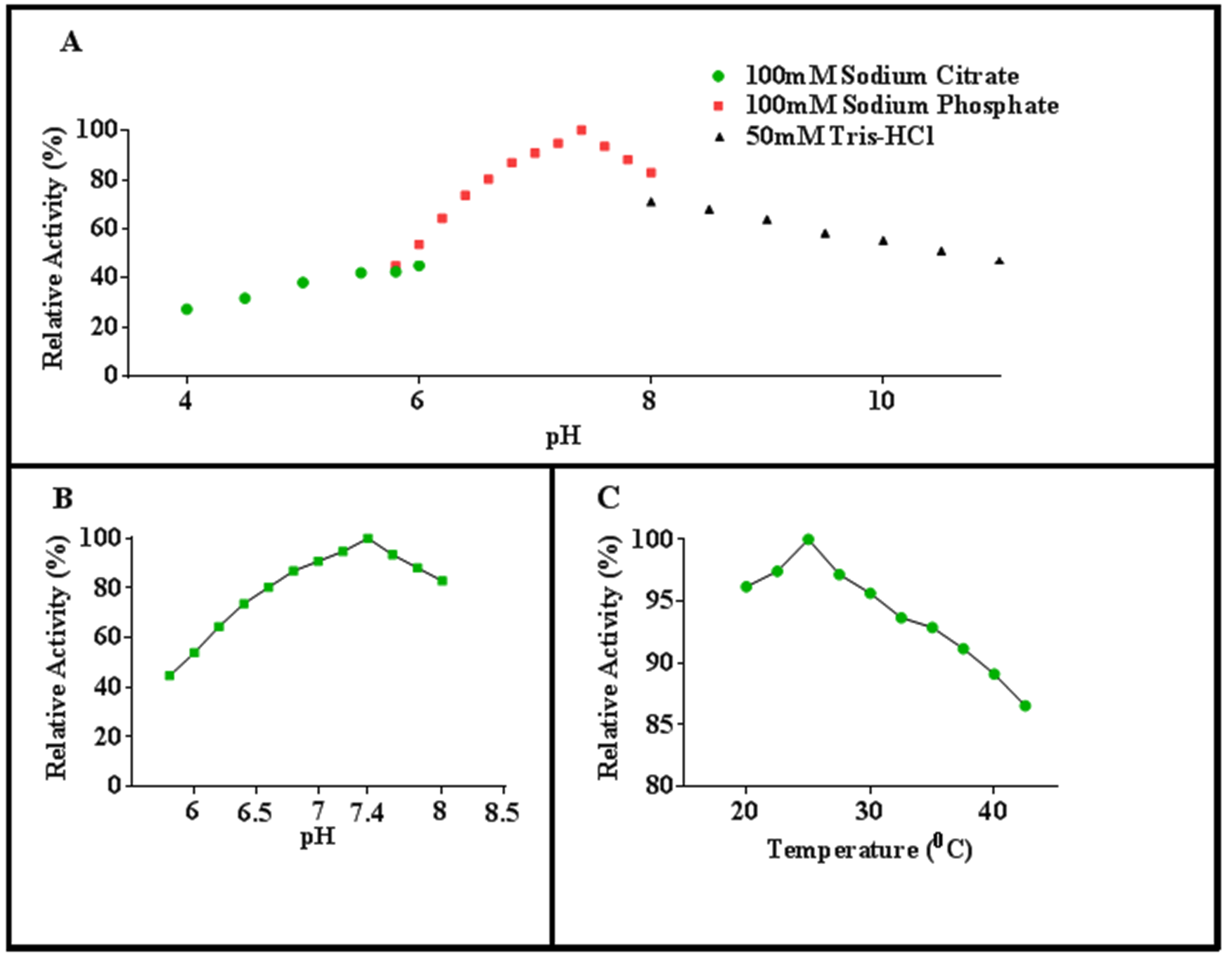

Supplement: S2 Fig — A. Relative activity of purified DHK in different buffer solutions (100mM sodium citrate pH 4.5–6.2, 100MM Sodium Phosphate pH 5.6–8.0 and 50mM Tris-HCl pH 7.8–11.5). The enzyme shows maximum activity at a pH of 7.4, it retains ~80% of its activity in buffer having a pH range of 6.8–8. B. Relative activity of DHK in 100mM sodium phosphate buffer previously shown to give optimal enzyme activity in varying pH range. C. The optimum temperature of the enzyme. The enzyme has highest activity at 25°C and loses most of its activity beyond 32°C. (TIF) [file pone.0170202.s002.tif]

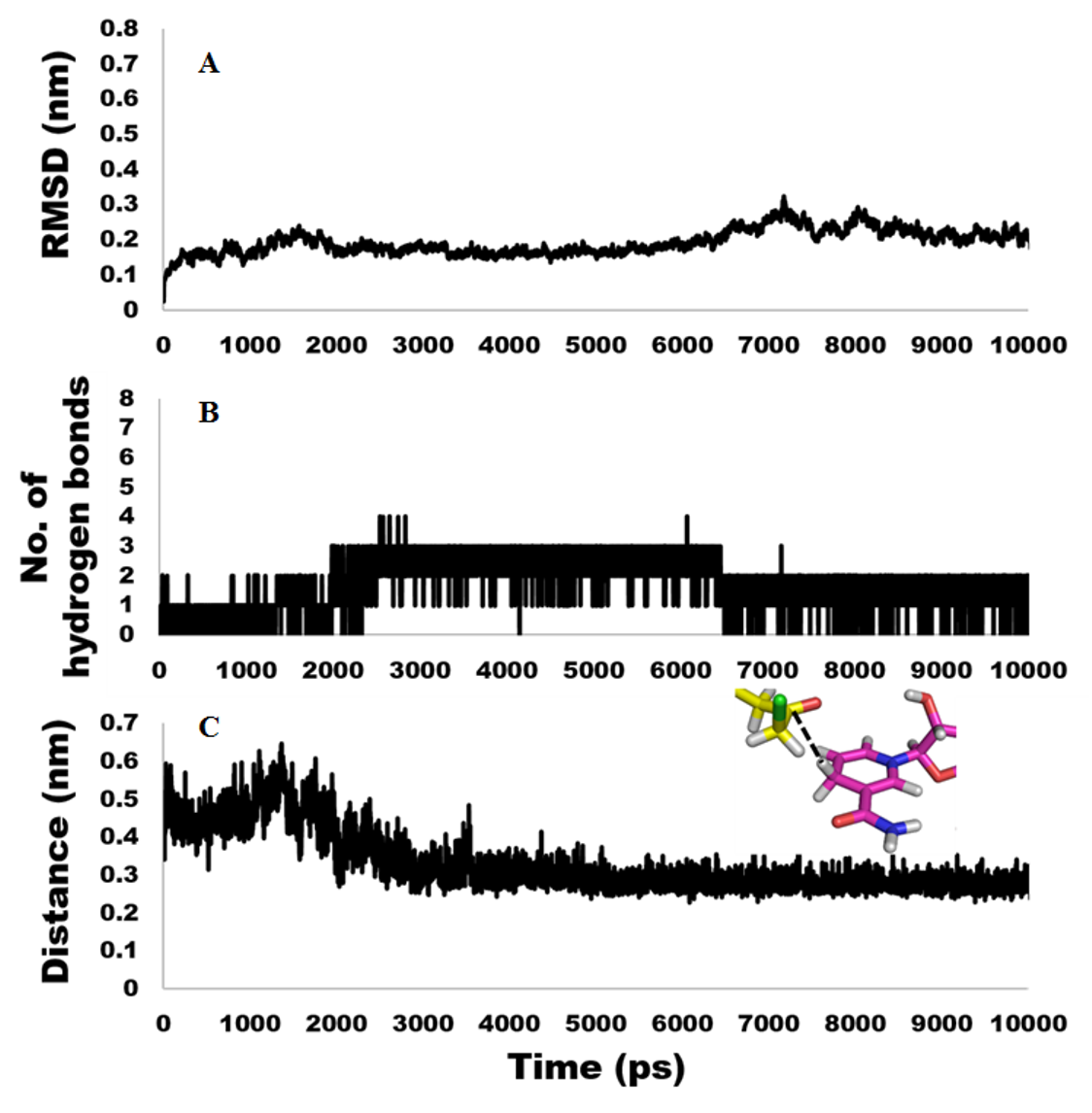

Supplement: S3 Fig — Backbone RMSD (A), No. of hydrogen bond interactions between substrate and DHK (B) and distance between substrate Carbonyl carbon atom and NADPH hydrogen (C) shown as line graph with respective to simulation time. (TIF) [file pone.0170202.s003.tif]

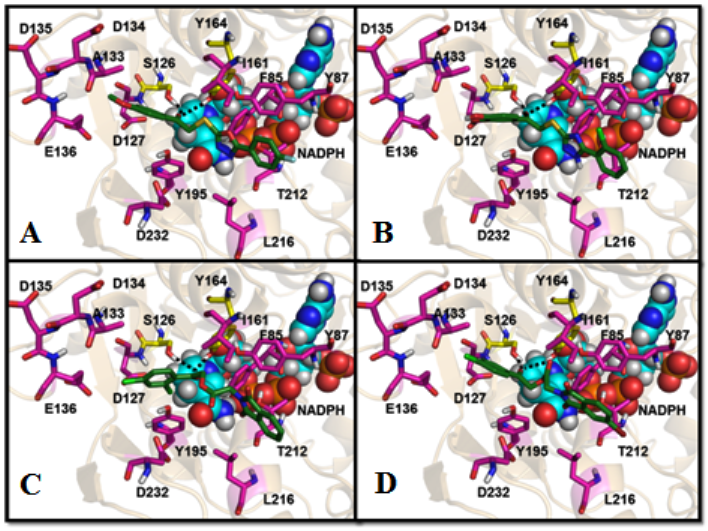

Supplement: S4 Fig — Molecular docking predicted binding modes of Compound 161 (A), Compound 219 (B), Compound 260 (C) and Compound 295 (D) with DHK protein. Active site, catalytic triad residues are highlighted with magenta and yellow color sticks respectively. Substrate molecules highlighted as green sticks and NADPH shown as cyan spheres. (TIF) [file pone.0170202.s004.tif]

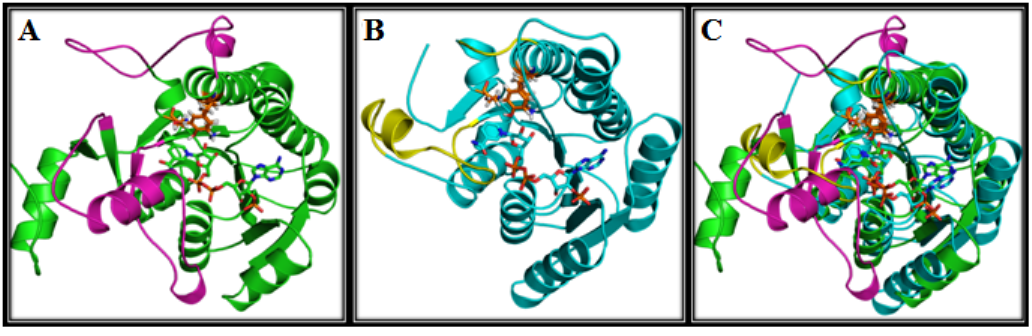

Supplement: S5 Fig — Structural comparison of DHK and FabG to understand the differences in substrate binding pocket. DHK (A), FabG (B) shown as green and cyan cartoons accordingly and superposed (C). Catalytic triad shown as orange sticks.Loops highlighted as magenta and yellow color on DHK and FabG structures accordingly to highlight DHK is having relatively longer loops. (TIF) [file pone.0170202.s005.tif]
